# Pepino (*Solanum muricatum*) Metabolic Profiles and Soil Nutrient Association Analysis in Three Growing Sites on the Loess Plateau of Northwestern China

**DOI:** 10.3390/metabo12100885

**Published:** 2022-09-20

**Authors:** Zhu Sun, Lihui Wang, Guangnan Zhang, Shipeng Yang, Qiwen Zhong

**Affiliations:** 1 Qinghai Key Laboratory of Vegetable Genetics and Physiology, Agriculture and Forestry Sciences Institute of Qinghai University, Xining 810016, China; 2 Laboratory for Research and Utilization of Germplasm Resources in Qinghai Tibet Plateau, Xining 810016, China; 3 College of Life Sciences, Northwest A&F University, Xianyang 712100, China

**Keywords:** pepino, metabolome, soil nutrients, association analysis

## Abstract

Different soil nutrients affect the accumulation characteristics of plant metabolites. To investigate the differences among the metabolites of pepino grown in greenhouses on the Loess Plateau in northwest China, we investigated the main soil nutrients and their correlation with metabolites. A total of 269 pepino metabolites were identified using UPLC-QTOF-MS to detect metabolites in fruits from three major pepino growing regions and analyze their differential distribution characteristics. A total of 99 of these substances differed among pepino fruits from the three areas, and the main classes of the differential metabolites were, in order of number: amino acids and derivatives, nucleotides and derivatives, organic acids, alkaloids, vitamins, saccharides and alcohols, phenolic acids, lipids and others. An environmental factor analysis identified soil nutrients as the most significant differentiator. Five soil nutrient indicators: TN (total nitrogen), TP (total phosphorus), AP (available phosphorus), AK (available potassium), and OM (organic matter), exhibited significant differences in three growing sites. Metabolite and soil nutrient association analysis using redundancy analysis (RDA) and the Mantel test indicated that TN and OM contributed to the accumulation of amino acids and derivatives, nucleotides and derivatives, and alkaloids while inhibiting organic acids, vitamins coagulation biosynthesis. Moreover, AP and TP were associated with the highest accumulation of saccharides and, alcohols, phenolic acids. Consequently, differences in soil nutrients were reflected in pepino metabolite variability. This study clarified the metabolite variability and the relationship between pepino and soil nutrients in the main planting areas of northwest China. It provides a theoretical basis for the subsequent development of Pepino’s nutritional value and cultivation management.

## 1. Introduction

Pepino (*Solanum muricatum* Aiton) is native to the northern highlands of the Andes in South America and is a member of the Solanaceae family [1,2]. It has juicy berries that vary in shape and color in its different varieties cultivated. The main cultivars have golden yellow skin covered with purple stripes at maturity and yellow flesh with an aromatic, slightly sweet taste and juicy characteristics [3,4]. Pepino can be consumed in various ways, in fruit and spinach salads similar to green and cooked vegetables, as a fresh fruit dessert, fruit juice, or puree [5]. The pepino is rich in minerals and vitamins such as thiamine, niacin, riboflavin, ascorbic acid, and Vitamin C. It is characterized as a functional food with high antioxidant activity [6,7]. Due to its low sugar content, pepino is recommended fruit for people with diabetes and is included in low-carb diets [8].

Currently, pepino is increasingly cultivated worldwide and has been introduced in Chile, Spain, Israel, New Zealand, and China [9,10,11]. Breeders and growers in different regions have developed and planted new Pepino varieties that are suitable for the local growing environment; for example, Spain has developed two pepino varieties, “Sweet Long” and “Sweet Round”, which are suitable for the Mediterranean climate [12]. In China, pepino is widely grown as a solanaceous fruit in Qinghai, Gansu, and Yunnan [13,14]. Pepino LOF (light-oval fruit) varieties are mainly grown in overwintering greenhouses in the Loess Plateau region of northwest China, mainly in Qinghai and Gansu. 

Based on market feedback, the same plant varieties grown in different areas showed different fruit metabolite accumulation patterns [15,16,17]. This is probably a result of many factors, including human management, irrigation, soil nutrients, and other environmental factors that can cause differences in fruit growth and development [18,19]. Different soil and available nutrients will contain different amounts of mineral nutrients and metabolites, impacting plant quality. Among them, nitrogen, potassium, and phosphate are vital for plant growth [20]. 

In plants, nutrients such as potassium, phosphorus, and nitrogen move from the older to the younger leaves during the vegetative stage or from leaves to seeds during reproduction [21]. The effect of nitrogen on metabolic substances was studied in *Lycium barbarum* fruits, and it was found that increased nitrogen contributed to the increase of amino acids as well as flavonoids in the fruit but inhibited polysaccharide production [22]. In pepino, the amount of nitrogen, phosphorus, and potassium fertilization affected the height, the number of leaves, days to flowering, and fruit yield [23]. Furthermore, the content of secondary metabolites in pepino fruits was affected by the amount of nitrogen, phosphorus and potassium fertilization [24].

Research on the association of soil nutrients with metabolites in crop production is currently in its infancy; however, metabolomics technologies are continuously improving. Developments in mass spectrometry metabolomics (MS) have revolutionized the discovery and annotation of chemicals from biospecimens on a large scale [25]. All living organisms contain small molecules, which can be isolated and quantified with metabolomics [26]. Such small molecules are responsible for many important properties of crop plants and their food products. Sugars, amino acids, fatty acids, and other nutritional ingredients are part of such chemicals, but also a myriad of ‘minor’ components, including phenolics, terpenoids, alkaloids, and others. These factors determine food quality and influence consumer perception and preferences [27]. In this study, we used a UPLC-QTOF-MS approach to examine the metabolites distribution profiles in mature pepino fruits from three major growing areas of pepino, HaiDong in Qinghai, WuWei in Gansu, and JiuQuan in Gansu. We additionally examined the physicochemical properties of the soil in these areas, aiming to investigate the association between the nutritional characteristics of pepino fruits with the soil indicators in different production areas. With this in mind, we provide theoretical guidance for pepino production requirements and cultivation management.

## 2. Materials and Methods

### 2.1. Sample Collection and Preparation

**Figure 1:**
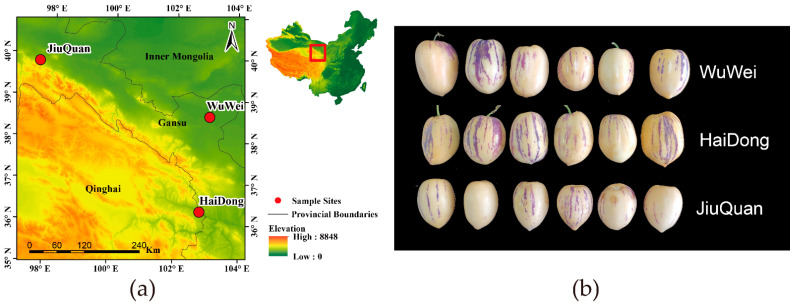
(**a**) Map of the sampling sites; (**b**) fruit phenotypes of pepino.

The sampling site (a) and fruit phenotype (b) are shown in Figure 1. The sampling sites were coded based on their location of sampling. The experimental materials for this study were greenhouse-grown large fruit types from three main growing areas: HaiDong (east longitude: 100°41.5′~103°04′, northern latitude: 35°25.9′~37°05′), Qinghai, WuWei (east longitude: 101°59′~103°23′, northern latitude: 37°23′~38°12′), Gansu JiuQuan (east longitude: 92°20′~100°20′, northern latitude: 38°09′~42°48′). These three areas were used to collect fruit and soil samples. Pepino samples from each regional source as a group, and each group were tested in six replicates. The collected fruits were immediately frozen in liquid nitrogen, then placed in a −80 °C freezer for subsequent studies in the laboratory. The soil was collected near the plants where the fruits were collected, with 0–20 cm of topsoil collected. The three regions were designated as three groups, and each group was tested in three replicates. All measurements were performed in the same laboratory.

### 2.2. Metabolomic Analysis

#### 2.2.1. Sample Processing

Six fruits from each region were cut into small pieces, mixed, and 50 mg was weighed and placed into 2 mL grinding tubes. An amount of 400 μL of pre-cooled 80% methanol was added for metabolite extraction in a total of six biological replicates with three technical replicates each. Subsequently, steel beads were added to the 2 mL grinding tubes, which were placed on a grinding instrument at a 50 Hz frequency at −20 °C for 60 s until the material was properly ground. This was followed by sonication in a water bath at 4 °C for 30 min, incubation at −20 °C for 30 min, and then centrifugation at 12,000× *g* for 10 min. An amount of 300 μL of the supernatant was transferred to another clean centrifuge tube. After another centrifugation at 12,000× *g* for 10 min, 150 μL of supernatant was transferred to the injection vial with a liner tube. All samples were mixed to make QC samples, and a total of four QC samples were made.

#### 2.2.2. UPLC-Q-TOF/MS

The UPLC-Q-TOF/MS characteristics and the protocol were the following: ACQUITY UPLC HSS T3 column (2.1 mm × 100 mm, 1.8 μm); mobile phase: 0.1% formic acid aqueous solution (A) −0.1% formic acid acetonitrile solution (B); gradient elution (0–3 min, 98–95% A; 3–7 min, 95–90% A; 7–8 min, 90–89% A; 8–20 min, 89–80% A, 20–23 min, 80–98% A, allowed re-equilibration of the column to initial conditions); column temperature: 30 °C; flow rate: 0.4 mL/min; Xevo G2-XS type UPLC-Q/TOF-MS liquid mass spectrometer, electrospray ionization source negative ion mode (ESI-), capillary voltage 2000 V, cone well voltage 40 V, ion source temperature 100 °C, desolvent temperature 400 °C, collision energy 10 V, desolventizing nitrogen flow rate 800 L/h, and cone hole backblast nitrogen flow rate 100 L/h. The desolventizing gas was nitrogen, and the collision gas was argon. The scanning range was 50–1500 m/z.

### 2.3. Metabolomic Data Analysis

The metabolome downstream data obtained were imported into the MS-DIAL software [28] for peak extraction, alignment, baseline calibration, deconvolution, and normalization. The positive and negative ion mass spectrometry data were downloaded from the MoNA (MassBank of North America, 2007 Free Software Foundation) database [29] to compare the identified metabolites. The following parameters were set as defaults: additive ions in the form of [M-H]^−^, [M-H]^+^.

### 2.4. Soil Nutrients (NPK) Measurements in the Soils of the Three Regions

Soil organic matter was measured using the potassium dichromate volumetric method [30]. The total nitrogen content was measured using the semi-micro Kjeldahl method [31], the total phosphorus content using the molybdenum antimony anti-colorimetric method [32], and the total potassium content with the aqua regia acid fusion-flame photometer method [33]. The available nitrogen content was measured with the alkaline diffusion method [34], the available phosphorus content with the hydrochloric acid-ammonium fluoride method [35], and the available potassium content was estimated by the ammonium acetate leaching-flame photometric method [36]. The pH was measured with the potentiometric method [37].

### 2.5. Statistical Analysis

The statistical analyses were carried out on the MetaboAnalyst 5.0 online platform [38]. The metabolites data were standardized by the following MetaboAnalyst 5.0 statistical analysis parameters: (1) Missing value processing: elements with missing values > 80% were removed, and the missing values were replaced by 1/5 of min positive values of their corresponding variables. (2) Data filtering was carried out using median absolute deviation (median absolute deviation (MAD)). (3) Data normalization (i.e., sample normalization, data transformation, and data scaling): Data normalization was performed by “normalization by the median”, “log transformation (base 10), and “auto-scaling (mean-centered and divided by the standard deviation of each variable).”

Analysis of variance (ANOVA) and partial least squares discriminant analysis (PLS-DA) was applied to discriminate metabolome differences among the three regions. The significantly different metabolites were designated based on the variable importance in the projection (VIP > 1) and *p* ≤ 0.05. We used MetaboAnalyst 5.0 to construct the DSPC network (Debiased Sparse Partial Correlation) and filter the network for betweenness > 100. A significant difference in soil nutrients between the three areas was determined by *p* ≤ 0.05. RDA and Mantel tests explored the relationship between metabolite classes and soil properties. Principal component analysis (PCA), stacked plots, and clustered heat maps were performed in R (version 4.1.3, John Chambers and colleagues, USA). Differences in soil properties were analyzed and visualized by Graph Pad software (Prism 9), and sampling maps were created using ArcGIS (version 10.2, ESRI, USA).

## 3. Results

### 3.1. Metabolite Analysis

**Figure 2:**
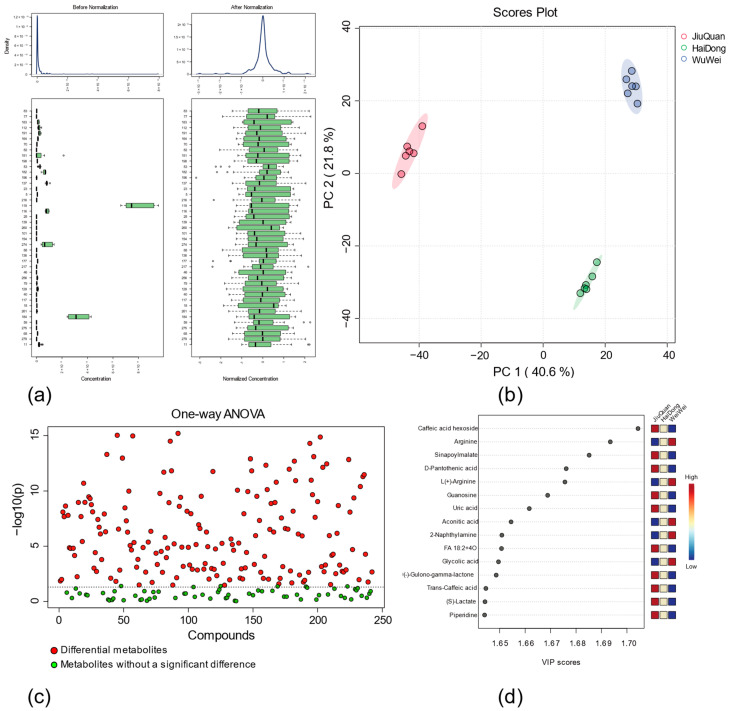
Integrated quality control of Metabolite analysis. (**a**) Metabolite normalization results; (**b**) metabolite principal component analysis, with samples from three regions divided into groups according to the source; (**c**) ANOVA analysis of differential metabolites; (**d**) the top 15 metabolites with VIP values greater than one from the PLS-DA analysis results.

This study investigated and measured the pepino fruit metabolite classes and their relative contents in three regions of Qinghai HaiDong, Gansu JiuQuan, and Gansu WuWei. The metabolome raw data were compared with the MoNA positive and negative ion mass spectrometry database (http://massbank.us/, accessed on 26 March 2022) in MS-DIAL, and 119 (pos) and 163 (neg) metabolites were identified, respectively. A total of 269 metabolites were finally identified (Table S1). The 269 metabolites were standardized, with the normalized results shown in Figure 2a. Applying an unsupervised PCA model, the first two components of PCA explained 40.6% and 21.8% of the variance, respectively. Results from principal component analysis (PCA) are presented in Figure 2b. The three sample groups (from a total of 18 independent pepino fruit samples) were clustered according to the region of origin. This indicated that the significant differences in metabolite levels among fruits resulted from the differences in the three cultivation regions. The six samples collected from each region were located in close proximity, indicating good reproducibility within each group. The four MS datasets of quality control (QC) samples were clustered together, indicating that the variation caused by the error of the detection instrument is small (Figure S1).

We compared the ionic intensity of each metabolite from the samples from different growing regions to detect the types of pepino fruit metabolites and differential metabolites in the three growing regions. The differential compounds in the three regions were separated based on the variable importance in projection (VIP), using partial least squares discriminant analysis (PLS-DA) analysis. A VIP score > 1 in PLS-DA was considered the cut-off to differentiate the metabolites, and a total of 99 were obtained (Figure 2d). Analysis of variance (ANOVA analysis) was used as another criterion to discriminate differential metabolites, and a total of 177 metabolites out of 269 metabolites were found to be different (Figure 2c). A total of 99 different metabolites met both the criteria of VIP > 1 and *p* ≤ 0.05 (Table S2).

### 3.2. Differential Metabolite Analysis 

**Figure 3:**
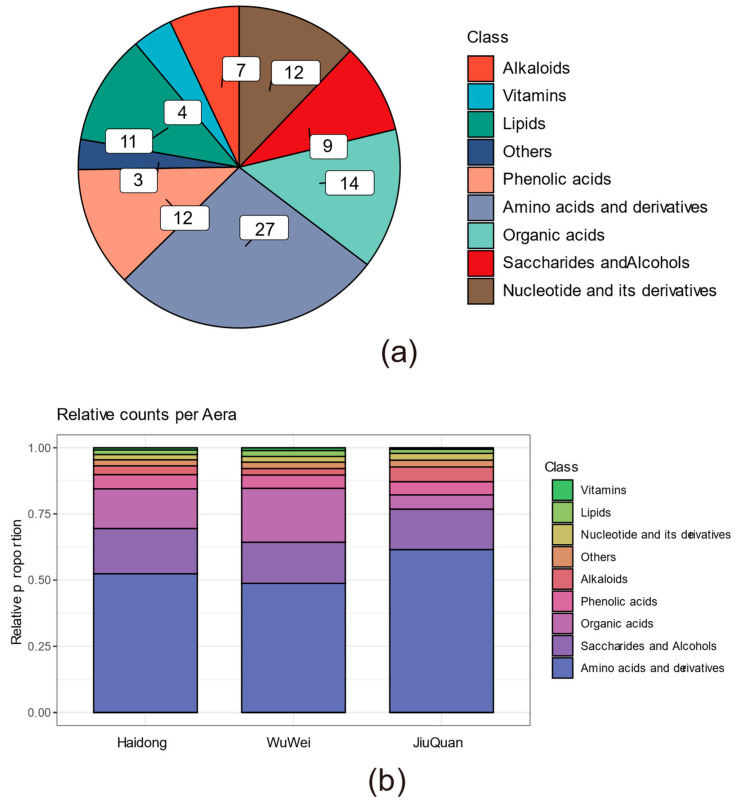
Classification of differential metabolites. (**a**) Pie chart showing differential metabolite classification. (**b**) The difference in the relative content of each metabolite class. The relative content of each metabolite class is summed by the content of the individual metabolites in them.

To better understand the variation in ionic strength of pepino fruit metabolites in the three growing regions, we divided the 99 differential metabolites into major categories (in total) and compared them (Figure 3a). These included 27 amino acids and derivatives, 12 phenolic acids-like substances, 12 nucleotides and derivatives, 7 alkaloids, 9 saccharides and alcohols, 4 vitamins, 14 organic acids, 11 lipids and 3 categorized as others.

The metabolite relative content analysis included all UPLC-QTOF-MS data (after normalization). Each metabolite’s relative content also differed significantly (Figure 3b). The relative content of the amino acids and derivatives was the highest in the three regions, corresponding to 50% of the total metabolites, and varied significantly among the three regions. The content of the amino acids and derivatives in the HaiDong region was intermediate. The phenolic acids category differed significantly, in contrast to the accumulation of Amino acids and derivatives in the three regions. They had the highest concentrations in the WuWei area, which were threefold higher than the JiuQuan area, followed by HaiDong and JiuQuan areas, respectively. The remaining classes of metabolites also differed, but the total amounts of those metabolites were less than 20% of the total metabolite content. The relative content of the nine specific metabolites in the validation set is shown in Table S3.

**Figure 4:**
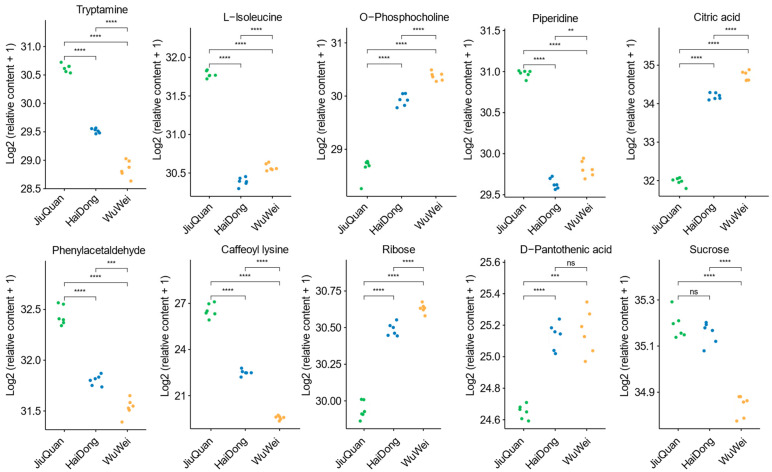
Compounds with the most significant differences in each category of differential metabolites. *p* > 0.05 (ns), *p* < 0.01 (**), *p* < 0.001 (***), *p* < 0.0001 (****). Sucrose was second only to ribose in terms of differences in sugars and alcohols but was the main sugar metabolite in pepino.

Statistical analysis was performed for the nine metabolites from nine classes with the most significant differences between the three regions. It is worth mentioning that sucrose among the saccharides and alcohols ranked second in terms of differences between the three regions. Still, sucrose is the most predominant sugar in pepino fruit, and we also analyzed it statistically. The results of the analysis for each metabolite are shown in Figure 4. In general, nine metabolites showed highly significant differences between regions, except for D-pantothenic acid (vitamins), indicating that the vitamins differed less between the three regions than other metabolites. Tryptamine, L-leucine, piperidine, phenylacetaldehyde and caffeoyl lysine were significantly lower in the WuWei region compared to the JiuQuan region. On the other hand, fruits from the WuWei region had higher contents of O-phosphocholine, citric acid, and ribose but the lowest sucrose content. In contrast, the fruits from HaiDong had no significant increase or decrease in each metabolite for the above. 

**Figure 5:**
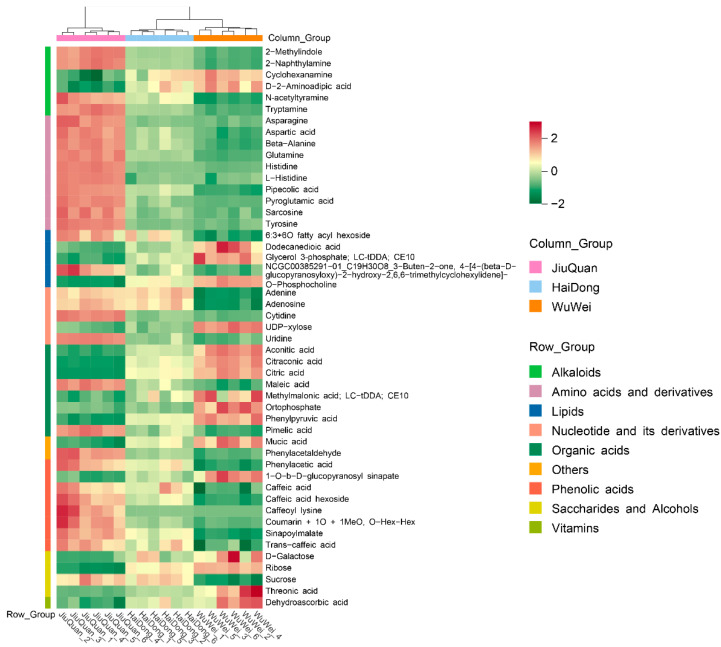
Clustering heat map of the top 50 differential metabolites.

The metabolites were analyzed by cluster analysis, and the top 50 metabolites with the most significant differences (VIP) among the 99 differential metabolites are illustrated with a clustering heat map (Figure 5). The metabolites differed significantly among the three regions, with fruits from JiuQuan being the most abundant. Alkaloids, amino acids and derivatives, nucleotides and derivatives, phenolic acids, organic acids, saccharides and alcohols, and vitamins were the most abundant. Saccharides, alcohols, and vitamins were higher in concentration in the fruits from WuWei. Lipids and other compounds were more abundant in the other two regions. Finally, the HaiDong region consistently exhibited an intermediate concentration level of each metabolite compared to the samples from JiuQuan and WuWei.

**Figure 6:**
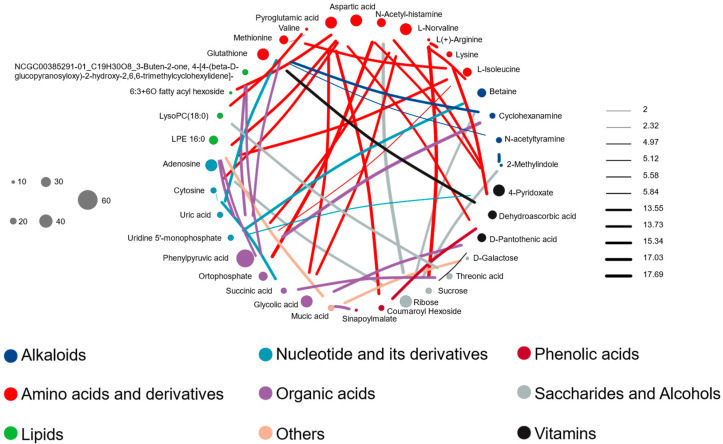
Differential metabolite correlation network. The size of each node represents the connectivity of the metabolite with the rest of the metabolites; the larger the connectivity, the larger the node. A coefficient represents the connectivity between metabolites; the larger the coefficient, the thicker the connectivity.

We developed co-accumulation patterns between pepino differential metabolites to refine the metabolites and characterize the pathways crucial in pepino metabolism. Metabolites that showed differential expression (76) were selected to elucidate potential interaction. After filtering out the betweenness of less than 100 linked metabolites, 36 differential metabolites were selected to infer a metabolic network (Figure 6). In the metabolite-related network, the node’s size represents the connectivity of the metabolite with the rest of the metabolites, and a coefficient represents the linkage between metabolites. The network indicated that amino acids and their derivatives played an important role. In addition, organic acids were closely linked with lipids, alkaloids, vitamins, sugars and alcohols, and phenolic acids. Sugars and alcohols are more likely to influence the content of lipids and alkaloids. The developed network identified associations between pepino metabolites based on their cluster level and further aided in redefining interactions between metabolites and gaining novel insights into their interactions.

### 3.3. Soil Nutrient Characteristics in the Three Planting Sites

**Figure 7:**
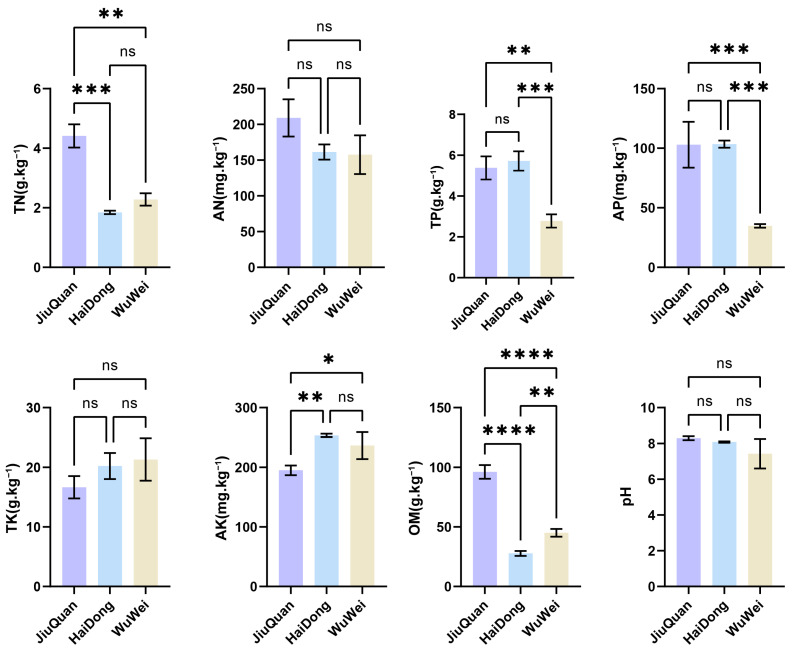
Differences in soil nutrients between the regions, *p* > 0.05 (ns), *p* < 0.05 (*), *p* < 0.01 (**), *p* < 0.001 (***), *p* < 0.0001 (****).

The initial analysis was conducted on soil samples collected from replicate plots at three pepino planting sites. The nutrient levels were highly variable among the sites and their corresponding soil types. The three analyzed groups differed for the soil nutrient indicators, such as the soil TN, TP, AP, AK, and OM contents. AN (available nitrogen), TK (total potassium) and pH have no significant differences were observed in soil (*p* > 0.05) (Figure 7). The TN and OM contents in JiuQuan soils were significantly higher compared to those in HaiDong (*p* = 0.0007, *p* < 0.0001) and WuWei (*p* = 0.0021, *p* < 0.0001). Compared with WuWei, HaiDong was characterized by higher TP (*p* = 0.0006) and AP (*p* = 0.0007); however, it did not exhibit significant differences when compared to JiuQuan. AK content did not significantly differ between HaiDong and WuWei, while it was significantly higher in both compared to JiuQuan (HaiDong, *p* = 0.0053; WuWei, *p* = 0.0255). Overall, the difference in soil nutrients between the three pepino planting sites was statistically significant, with the highest soil nutrients found in the JiuQuan area (TN, TP, AP, AK, and OM).

### 3.4. Correlation Analysis of Soil Properties and Differential Metabolites

**Figure 8:**
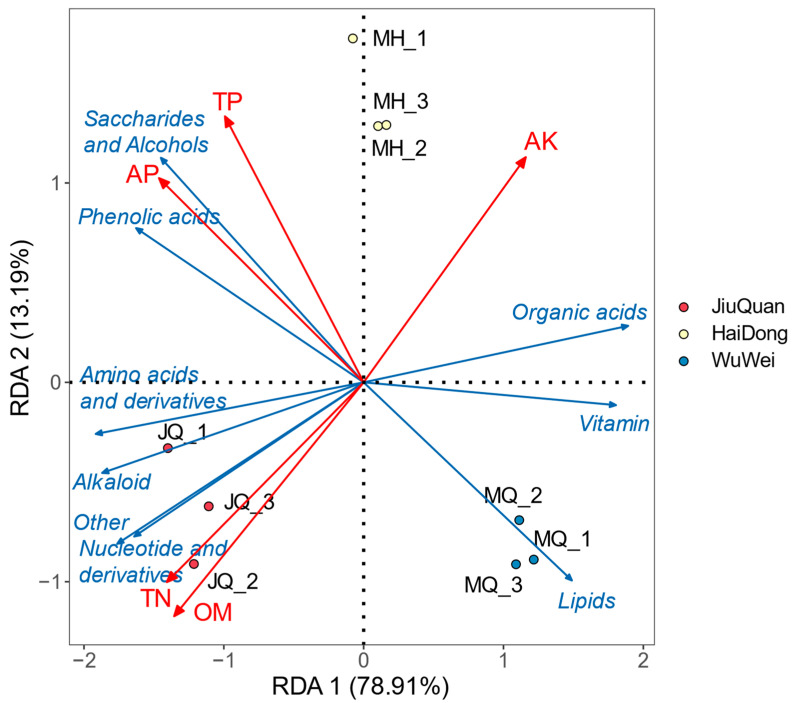
RDA plots of metabolites related to soil physicochemical properties. Yellow: HaiDong (MH1-3); red: JiuQuan (JQ1-3); blue: WuWei (MQ1-3). The angle between soil physicochemical properties and metabolites indicates the correlation magnitude. The smaller the angle, the higher the correlation and vice versa.

A redundancy analysis (RDA) showed that the two primary coordinates explained 92.1% of the variance (78.91% for the first axis and 13.19% for the second axis). In addition, the RDA ordination biplot illustrated the impact of soil nutrients of the different planting sites on metabolite accumulation in pepino (Figure 8). The differences observed were statistically significant (F = 10.11, *p* < 0.005). Specifically, a higher concentration of Amino acids and derivatives, alkaloids, nucleotides and derivatives, and other compounds were present in the fruits from JiuQuan. These compounds were positively correlated with TN and OM, while a negative correlation was observed with organic acids and vitamins. Saccharides, alcohols, and phenolic acids exhibited higher positive correlations with AP and TP and significant negative correlations with Lipids. Only lipids, vitamins, and organic acids were higher in the fruits of the WuWei area.

**Figure 9:**
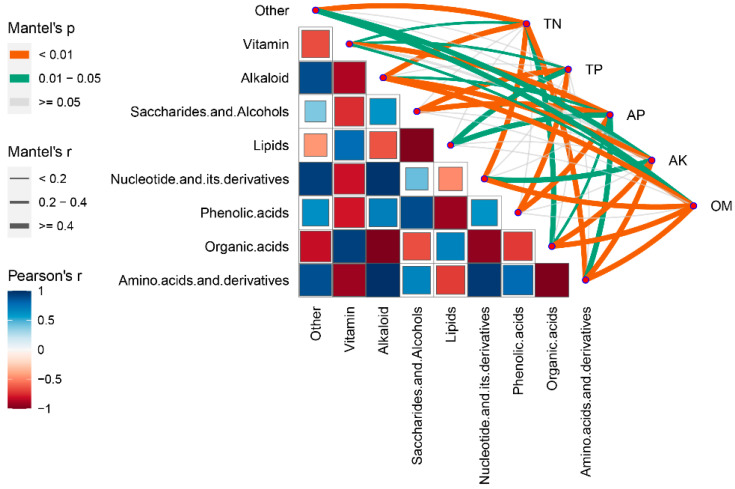
A complex Pearson’s correlation matrix illustrating metabolite-metabolite and soil nutrients correlations across populations. To construct the complex correlation matrix, metabolite-metabolite (heatmap) and soil nutrients (lower links) were log2-transformed. A box inside the heatmap indicates a correlation. Blue and red represent Pearson’s r value, respectively. Links with *p*-value and |r| value show the correlation between metabolites and soil nutrient traits. The width of the edges indicates the discrete Mantel’s r, and the orange and green colors indicate Mantel’s p, respectively.

Analyzing the total abundance of nine classes of metabolites (Figure 9), the Pearson correlation analysis indicated that amino acids and derivatives (r = 0.989, *p* < 0.001), Nucleotides and derivatives (r = 0.971, *p* < 0.001), and compounds classified as others (r = 0.891, *p* = 0.001) showed a significant positive correlations with alkaloid-like substances. A significant negative correlation was observed between Organic acids (r = −0.986, *p* < 0.001) and Vitamin (r = 0.879, *p* = 0.002). Saccharides and alcohols (r = 0.891, *p* = 0.001) substances were positively correlated with phenolic acids, and negatively correlated with lipids (r = −0.920, *p* < 0.001). This led us to classify the nine metabolite classes into two broad categories. The first consisted of amino acids and derivatives, nucleotides and derivatives, alkaloids, organic acids, vitamins, and other compounds. The Mantel test analysis of soil data and metabolite classification data showed that the accumulation of compounds in the first category significantly correlated with the content of TN, OM, and AK (*p* < 0.01). In contrast, the second major category of metabolites, including saccharides and alcohols, phenolic acids, and lipids, correlated more with TP and AP (*p* < 0.05). This is consistent with the results of the RDA analysis.

## 4. Discussion

We analyzed the LOF type pepino metabolome in the Chinese Loess Plateau collected from three planting sites. The chemical components’ type and concentration in the fruit were evaluated. The relationship between the fruit metabolites accumulation pattern and soil conditions, i.e., the soil nutrient conditions required to accumulate specific nutrients in the fruit was discussed.

Due to the different cultivation environments, pepino fruits of the same variety differ significantly in size and chemical composition [39]. This leads consumers to prefer fruits from specific regions and to choose high-quality foods with clear geographical characteristics when purchasing. At the same time, producers can gain more market recognition for their unique growing regions [40]. Thus, there is a need to explore the key chemical components that cause differences in pepino metabolites across regions and the environmental conditions that influence this component. This will help to better differentiate pepino fruit from different production regions and for producers to improve the available fruit chemistry to meet consumer purchasing needs [41].

We identified significant differences in the chemical composition of the fruits from the three production areas. The non-targeted metabolomics approach revealed a total of 99 differential metabolites detected in all samples, classified into nine known classes. Previous studies reported that pepino fruit sugars consist of sucrose, fructose, and glucose, with sucrose accounting for 50% [42]. They contain non-volatile Organic acids such as citric acid, malic acid, and quinic acid, with citric acid being the major Organic acid [43]. pepino additionally contains many amino acids, with aspartic acid being the most abundant, accounting for 70% of the total free amino acids. Vitamin levels are higher than the typical levels found in most fruits [44,45]. Glucose and sucrose were detected in this study, and sucrose was considered a metabolite that differed between regions. This corroborates that sucrose is the predominant sugar in pepino fruits, and it is hypothesized that the variation in sugar content in pepino fruits is mainly caused by sucrose [46]. Many organic acids were identified at the same time, and citric acid was the most varied substance among organic acids with the highest content [47,48], which is consistent with the previous studies.

The metabolites of *Lycium barbarum* fruit from the main growing regions of northwest China were compared, with the metabolite content varying significantly among growing regions [49]. Shi et al. [50] performed a metabolomic analysis on dried dates from seven different geographical sources, which their phytochemical composition could clearly distinguish.

Most of the differential metabolites detected could distinguish pepino fruits from the different growth environments. Fruits collected from JiuQuan contained more Alkaloids, Amino acids and derivatives, Nucleotides and derivatives, Saccharides and Alcohols, and phenolic acid. On the other hand, Organic acids and Vitamins were more abundant in the fruits of WuWei, while the fruits of HaiDong always had an intermediate concentration level of those metabolites. Therefore, the pepino sampling areas of this study, HaiDong City, Qinghai Province; JiuQuan City, Gansu Province; and WuWei City, Gansu Province, could be distinguished according to the different metabolite types and contents. The significant differences in the levels of metabolites in fruits grown in other geographical areas were due to several factors. The main contrast in these areas was the soil’s TN, TP, AP, AK, and OM content. 

Previous studies have pointed out the effect of soil properties on metabolite composition. Radi et al. [51] showed that when N fertilizer was applied, it lowered the P and K content resulting in lower concentrations of phenolic compounds and soluble sugars. Interestingly the concentration of Organic acids would decrease with the increased amount of N fertilizer applied. These findings are in agreement with the results of our study. N fertilizers can affect the ratio of soluble sugars to titratable acidity, and the combination with NPK fertilizers favors the increase of total phenolic content, soluble sugar content, and antioxidant activity of fruits, as well as inhibits the biosynthesis of organic acids in fruits [52,53]. Low doses of potassium fertilizer resulted in fruits with lower polyphenol and sugar content [54]. 

In contrast, our results indicate that the phosphorus content of the soil influence to a greater extent, the synthesis of phenols and sugars in pepino. It could be due to the fact that we are calculating the relative content of all phenols and sugars in pepino, whereas, in previous studies, only one or a few representative substances were measured. Therefore, the metabolite contents of pepino need to be measured quantitatively in subsequent studies to obtain more accurate results. The organic acids and soluble sugars content are the most representative indicators of fruit quality and taste [55,56,57]. The increase in phenolic substances as well as vitamin-like antioxidant compounds is another indication of improved fruit quality [58,59]. In general, the JiuQuan soils contained the highest TN and OM contents and TP and AP contents, significantly higher than those of the WuWei area but not significantly different from those of the HaiDong area. Accordingly, the fruits collected from JiuQuan contained a higher concentration of phenolic acids, amino acids and their derivatives, and sucrose, the most important sugar in pepino.

On the contrary, fruits from WuWei accumulated more vitamins, organic acids, and other compounds. The soil’s TN and OM contents promoted the accumulation of amino acids and derivatives and nucleotides and derivatives. They inhibited the production of organic acids and vitamins in pepino fruits. In contrast, AK facilitated the production of organic acids and vitamins.

**Figure 10:**
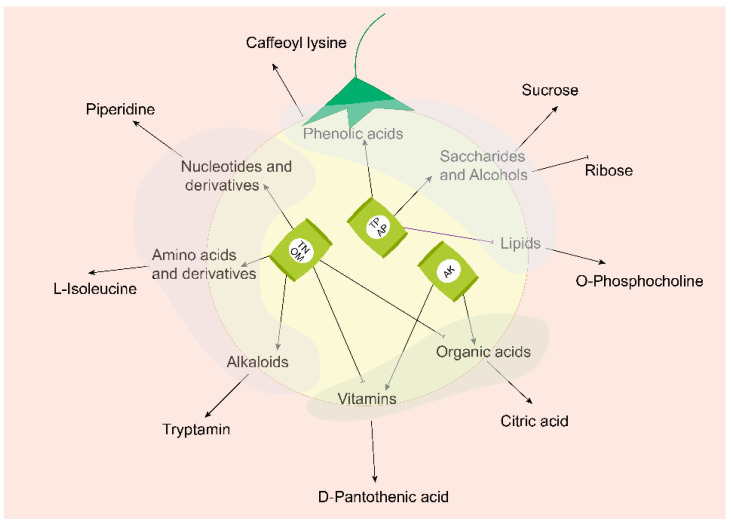
Effect of soil nutrients on pepino metabolic compounds.

On the other hand, the presence of P in the soil strongly correlated with Saccharides, alcohol substances, and phenolic acid accumulation in the fruits. Both AP and TP contributed to the accumulation of saccharides, and alcohol substances and phenolic acids. At the same time, the production of Lipids demonstrated an opposite pattern (Figure 10). The higher overall soil content of N, P, and OM in the JiuQuan area resulted in a higher content of most metabolic compounds in the fruit. In the WuWei area, interventions might be needed to increase the content of the substances mentioned above in the soil and thus increase the content of the corresponding metabolic compounds. Therefore, applying N, P, and K in combination with a targeted increase of N and P is an effective way to increase the favorable metabolic compounds in pepino fruits. However, we only collected fruits and soils during our study’s ripening stage. This represents the results of the differences in fruit metabolites and the effect of soil conditions only at a single stage. Additional non-targeted metabolomics approaches should be further employed, as many more undetected compounds may exist. Therefore, further studies are needed to detect the continuous changes of metabolites during the growth and development of pepino, providing the whole picture of the effects of soil conditions at every developmental stage. This will be instrumental in standardizing and improving the process of pepino cultivation and growth.

## 5. Conclusions

This study assessed the metabolite composition of pepino fruit and the soil environmental factors that might affect metabolite formation in pepino fruit. LOF-type pepino varieties widely grown in northwest China’s main pepino growing areas were collected from three typical growing areas. The results of the non-targeted metabolomics assays showed that pepino produced in JiuQuan, WuWei and HaiDong regions contained significantly different metabolites, and a total of 99 differential metabolites were detected and classified into nine known categories. The pepino from different regions can be distinguished according to the differences in metabolites, among which amino acids and derivatives, organic acids have the most significant differences, while vitamins have the least content and the smallest differences. 

Based on the results of correlation analysis between differential metabolites and soil nutrient indicators, we found that soil environmental factors had a significant effect on fruit chemical composition. In pepino cultivation, applying nitrogen fertilizer increased the content of amino acids and derivatives, alkaloids, and nucleotides and derivatives in the fruit. pepino fruit with higher sugar content requires more phosphorus fertilizer, while fruit with more bioactive substances requires more potassium fertilizer. Therefore, different soil environmental factors can alter the metabolite composition and chemical content of pepino fruits, and a higher level of NPK in the soil will increase sugar content and antioxidant activity in fruits. Thus, growers will be able to produce better-tasting, higher-quality fruits.

## Data Availability

Data Availability Statements in the maintext.

## Supplementary Materials

The following supporting information can be downloaded at: www.mdpi.com/2218-1989/12/10/885/s1, Figure S1: QCA of QC samples with three regional pepino samples; Figure S2: Total insolation in the three areas; Figure S3: Net insolation in the three areas; Figure S4: Temperature in the three regions; Table S1: 269 identified metabolites; Table S2: 99 different metabolites; Table S3: The relative content of the nine specific metabolites groups. Documents S1: Raw data; Documents S2: Annotation data.
